# Comparison of the Systemic Lupus Erythematosus Activity Questionnaire and the Systemic Lupus Erythematosus Disease Activity Index in a Black Barbadian Population

**DOI:** 10.1155/2013/875369

**Published:** 2013-10-24

**Authors:** Kim R. Quimby, Cindy Flower, Ian R. Hambleton, R. Clive Landis, Anselm J. M. Hennis

**Affiliations:** ^1^Chronic Disease Research Centre, Tropical Medicine Research Institute, The University of the West Indies (UWI), Jemmott's Lane, St. Michael, Bridgetown BB11115, Barbados; ^2^Faculty of Medical Sciences, The University of the West Indies (UWI), Cave Hill Campus, St. Michael, Bridgetown BB11000, Barbados

## Abstract

In Barbados, use of the Systemic Lupus Erythematosus (SLE) Disease Activity Index (SLEDAI) is limited by the unavailability of serologic markers. The SLE Activity Questionnaire (SLAQ) excludes laboratory measurements and is therefore more accessible. Here, we investigate the agreement between the SLAQ, the SLEDAI, and the physician global assessment (PGA). A pilot of 32 participants completed the SLAQ and SLEDAI. The tools were compared (1) in their original format, (2) limited to common indices, and (3) limited to the same patient recall period. We compared the proportions of persons reporting disease activity and the concordance between calculated activity scores for SLAQ versus SLEDAI and for SLAQ versus PGA. Seventy-eight percent versus 59% of participants reported disease activity with the original SLEDAI versus SLAQ, respectively. The relationship was reversed to 22% versus 59% when the matched item tools were compared. Concordance was 0.62 (95% CI 0.42–0.81) between the original scores, 0.70 (0.57–0.83) when restricted by matched items, and 0.72 (0.59–0.84) when further restricted by recall period. Concordance between the SLAQ and PGA was 0.56 (0.32–0.80). Reversal of the disease activity percentage in the matched items comparison highlights the inadequacy of tools that exclude laboratory measurements and suggests that the subjective nature of SLAQ may contribute to over-reporting. Further work is needed to produce a robust disease activity tool apt for resource-constrained environments.

## 1. Introduction

Systemic Lupus Erythematosus (SLE) is a multisystem autoimmune disease characterized by periods of clinical quiescence punctuated by acute disease flares. A five-year population-based study highlighted the striking disease excess among women of African descent, with crude incidence rates (per 100,000) of 0.4 for White males, 3.5 for White females, 0.7 for African-American males, and 9.2 for African-American females [1]. Mortality rates follow a similar pattern, with several studies indicating higher rates of lupus deaths among Black women compared to their Caucasian counterparts [2–5]. Some of the regions with the highest disease burden and mortality rates are also the least equipped to diagnose and manage the disease [6]. Barbados, a Caribbean nation with a population of 288,000 persons [7], 93% of African origin, has one of the highest documented incidence rates of SLE among women (12.21 per 100,000 person-years; 95% CI 10.46–14.18) [5]. Similar to the experience of African-Americans, SLE in Barbadian patients has been reported to run a clinically aggressive course, with a 5-year survival rate of 79.9% (95% CI 69.6–87.1). In this Barbados cohort, 47 percent of patients developed lupus nephritis, which reduced survival in this subpopulation to 68% (95% CI 51–80) [5]. Despite the documented clinical impact of lupus in Barbados, routine monitoring using internationally accepted disease activity indices such as the Systemic Lupus Erythematosus Disease Activity Index (SLEDAI) is not feasible for most patients, as two principal markers of disease activity, the complement levels and anti-dsDNA titres, are not routinely available.

Recommendations for SLE management developed by the European League Against Rheumatism (EULAR) and the American College of Rheumatology (ACR) focus on international best practice. However, the financial resources and available infrastructure in many countries do not allow for these guidelines to be achieved in practice [8, 9]. A report of an international symposium at the 9th International Congress on SLE in 2010 highlighted the disparities in the management of SLE worldwide and sought to establish a consensus on the minimum best practice guidelines which may be employed in resource-poor clinical settings [10]. The Systemic Lupus Activity Questionnaire (SLAQ) was considered a suitable screening tool which could guide the more in-depth assessment by the clinician.

The SLAQ is a self-administered questionnaire which has been developed to monitor disease activity in populations with financial constraints and has been compared to the Systemic Lupus Activity Measure (SLAM) with promising results [11]. This contrasts with its reported poor correlation with the SLEDAI and with the Physician Global Assessment (PGA) which is considered the best evaluation of disease activity [12]. Differing statistical methods used in previous questionnaire comparisons and the structural differences between questionnaires (SLAQ and SLEDAI, for example, use different patient recall times) hamper the ability to compare studies. In view of the possible application of the SLAQ, but its varying agreement with the other instruments, our aim was to assess the ability of the SLAQ to measure disease activity compared to the SLEDAI and the PGA, utilizing a single statistical measures of agreement and providing a simple sensitivity analysis of agreement by standardizing questionnaire items and questionnaire recall time.

## 2. Materials and Methods

Ethical approval was obtained from the Ministry of Health/University of the West Indies Ethics Review Board and the Queen Elizabeth Hospital Ethics Committee.

### 2.1. Participants

The Barbados Lupus Registry was established in 2007. In December 2009, there were 226 persons alive with definite SLE (ACR ≥ 4 criteria) of whom 98% were of Black ancestry and the majority (94%) were female. The 32 participants involved in this pilot were a convenience sample from this cohort selected for a companion investigation. Each participant completed the SLAQ, and a thorough systematic examination including administration of the SLEDAI was performed by the study rheumatologist.

### 2.2. Disease Activity Indices

#### 2.2.1. The Systemic Lupus Activity Questionnaire (SLAQ)

The SLAQ is a self-reporting tool that assesses the presence and severity of twenty-four clinical indices over the previous three months [11]. It carries a weighting regime identical to the SLAM with 0 points awarded for absent disease, 1 for mild, 2 for moderate, and 3 for severe disease, yielding a range from 0–44 points [11]. In addition, there is a single question assessing the presence and severity of lupus activity with a score ranging from 0 (no flare) to 3 (severe flare) and a single numerical rating of disease activity from 0 (no activity) to 10 (most activity).

#### 2.2.2. Systemic Lupus Erythematosus Disease Activity Index (SLEDAI)

The SLEDAI is a physician-administered instrument accounting for the preceding 10 days. It assesses 16 clinical features and 8 laboratory indices. The weight which has been applied to each index gives this tool a range of 0–105 points. All participants in this study completed the full SLEDAI including the dsDNA titres and the complement 3 levels.

#### 2.2.3. SLAQ versus SLEDAI

There are considerable similarities between the SLAQ and SLEDAI with 15 of the 24 SLAQ and 12 of the 24 indices of SLEDAI overlapping. These were (SLEDAI descriptor/SLAQ question) seizure/seizure, organic brain syndrome/forgetfulness, lupus headache/unusual headaches, CVA/stroke, vasculitis/white fingers or toes in cold, arthritis/joint pain or swelling or stiffness, myositis/muscle pain or weakness, new rash/other rash, alopecia/bald patches on scalp, mucosal ulcers/rash in mouth or nose, pleurisy/shortness of breath or chest pain on deep breathing, and fever/fever (Table 1).

#### 2.2.4. PGA

The PGA is based on the physicians' overall assessment of disease status. It is a composite of disease activity tools, other clinical or laboratory markers not included within the tools, and the physician's knowledge of the patient disease history. It carries the score of 0 for no disease, 1 for mild disease, 2 for moderate disease, and 3 for severe disease.

### 2.3. Statistical Analysis

Agreement between SLAQ and SLEDAI was assessed using the concordance coefficient for approximately continuous scores (SLAQ score, SLEDAI scores) or using Kendall's W agreement for ordinal scores (PGA, SLAQ flare). Agreement was assessed under various data restrictions, each designed to increase the clinical comparability of the two tools. Firstly, the tools were compared in their original state. Then, in a simple sensitivity analysis, agreement was further assessed (a) using only indices that were included in both SLAQ and SLEDAI—this reduced each tool to 12-items—and (b) analyzing responses from participants exhibiting symptoms within the SLEDAI 10-day participant recall window.

Agreement was also assessed between the SLAQ flare rating (i.e, the single question assessing the presence and severity of lupus activity) and the PGA.

## 3. Results

Fifty-nine percent of the 32 participants reported disease activity using the original SLAQ, compared to 78% identified by the SLEDAI. Based on the matched item scale, only 22% of participants reported activity with the SLEDAI whereas the SLAQ result did not change (59%).

**Figure 1:**
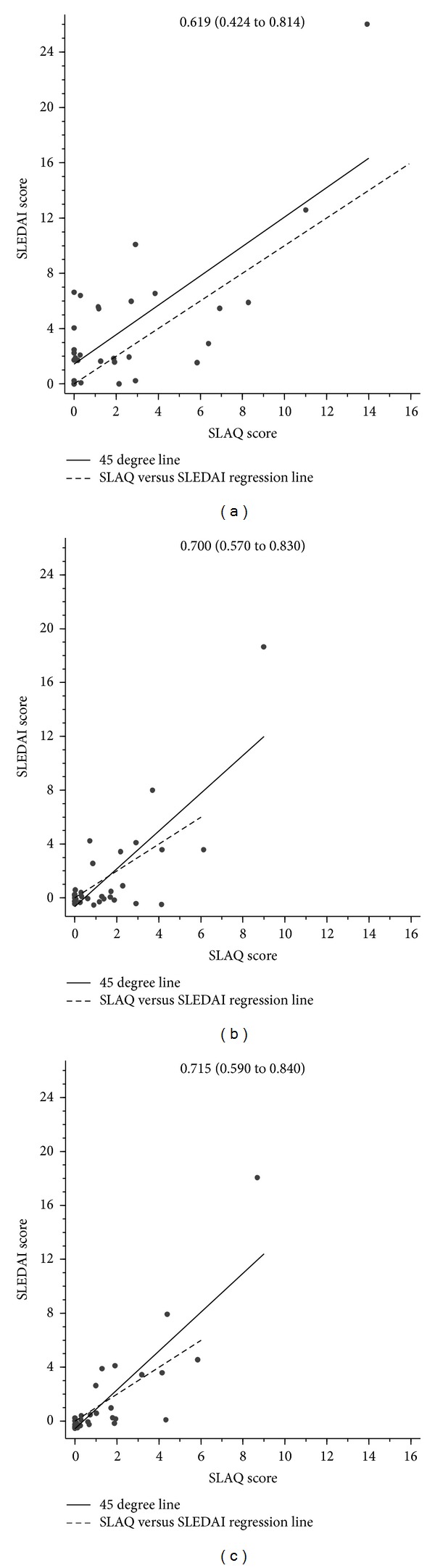
The concordance between (a) the original activity scores, (b) the matched-items score, and (c) the scores further restricted by recall time.

Concordance between the original activity scores was 0.62 (95% CI: 0.42 to 0.81) (Figure 1(a)). Restricting the tools to common items increased the concordance to 0.70 (0.57 to 0.83) (Figure 1(b)). Two participants experienced disease flares within the SLAQ but not the SLEDAI timeline. Reanalysis with the removal of the scores from these 2 patients further increased the agreement between the SLAQ and SLEDAI instruments to 0.72 (0.59 to 0.84) (Figure 1(c)).

Agreement between the SLAQ flare rating and the physician global assessment was 0.56 (0.32 to 0.80).

## 4. Discussion

The SLEDAI is widely accepted as a tool for monitoring SLE activity both in clinical practice and in research. However, the SLEDAI includes laboratory measures not routinely available in resource-constrained settings as experienced in Barbados, and therefore, the resulting clinical assessments are often incomplete. The SLAQ is a self-administered questionnaire that is inexpensive but gives variable results when compared to other instruments measuring lupus activity.

Our comparison of the SLAQ and SLEDAI instruments presented several challenges. Firstly, although both tools monitor lupus activity, they assess different disease domains and are therefore not expected to be in total agreement. For example, psychosis, visual disturbance, cranial nerve disorder, pericarditis, and laboratory markers are assessed by SLEDAI but not by SLAQ, whereas weight loss, fatigue, malar rash, lymphadenopathy, and photosensitivity are assessed by SLAQ and not by SLEDAI. To partly overcome this, an altered questionnaire which mapped only indices common to both was used (Table 1).

Secondly, the timeframe for patient symptom recall varies between instruments. The SLEDAI takes into account the previous 10 days whereas the SLAQ covers a 3-month period. Two participants experienced flares between the 10-day and 3-month window (measured by the SLAQ but not the SLEDAI). Exclusion of the scores of these 2 patients resulted in better agreement between the scores of the SLAQ and the SLEDAI (Figure 1(c)).

In spite of the usefulness of the disease activity measures, the PGA is still considered the best assessment of disease activity. Concordance between the SLAQ flare rating and the PGA was 0.56 (0.32 to 0.80), significantly lower than the SLAQ-SLEDAI scores concordance of 0.72 (0.59 to 0.84), indicating that a collection of multiple indices is more accurate than a single self-reported measure of disease activity over the previous three months.

Fifty-nine percent of participants reported disease activity with the SLAQ compared to 78% identified by the SLEDAI. This seemed to dispel the notion that the subjective nature of SLAQ leads to the over-reporting of symptoms; however, this difference could be attributed to the disparity in reporting laboratory indices. Laboratory indices are only assessed by the SLEDAI, and it is reasonable to expect that more indicators of disease would be identified by this method. To adjust for the inequity in the indices being assessed, we investigated these summary measures using the matched item scale. The SLAQ results did not change, with 59% of participants reporting disease activity. However, the proportion reporting disease activity now decreased to 22% (from 78%) with the reduced item SLEDAI. This indicated two things; firstly, it highlighted the inadequacy of disease activity indices that do not include laboratory measures; in this case, there was a 56% point reduction in the disease markers identified. All cases of proteinuria, which is critical to diagnosis of nephritis which affects nearly half of Barbadian patients, would have been missed. The mandatory inclusion of basic laboratory measures, for example, full blood count and urinalysis in the monitoring of SLE suggested during the 9th International Congress on SLE in 2010, was aimed at rectifying this gap [10]. Secondly, it underscored the difference in outcome between physician-rated and patient-reported conclusions, even when matched clinical indices are being compared. In this pilot, more disease activity was recounted with the SLAQ suggesting that its subjective nature might lead to over-reporting by patients who do the reporting. With the support of the Barbados Lupus Cohort [5], we intend to examine these potential deficiencies in a longitudinal comparison of the SLAQ and SLEDAI questionnaires, where both tools will be administered to participants at predetermined intervals. This follow-up study will allow for further investigations to identify particular domains of the SLAQ which promote over-reporting. With the additional statistical power in this longitudinal investigation, we will examine the agreement of individual matched questionnaire items from SLAQ and SLEDAI. Those SLAQ indices that exhibit low agreement with the matched SLEDAI indices would be flagged for deletion or substitution. Any SLAQ alterations would then be subjected to further validation.

Construction of this tool is even more relevant now with the thrust towards the treat-to-target initiative, which has proven to be beneficial in the management of rheumatoid arthritis [13, 14]. Using this method, clinical targets for each follow-up time-point is established, and at the time of follow-up, therapy is adjusted based on the realisation (or not) of the predefined disease activity goal. Measuring the identified target requires a relevant disease activity tool.

In conclusion, the current SLAQ is inadequate as a disease monitoring tool because of its lack of laboratory measurements and its patient-based subjective reporting which might lead to inadvertent over-reporting of symptoms. This leaves us still in need of a SLE disease monitoring tool which is accessible to resource-limited populations. The aim of our future study is to transform the current SLAQ into a clinically relevant but accessible tool by adding basic, cheap laboratory measurements and reducing over-reporting of symptoms.

## Figures and Tables

**Table 1:** 

SLEDAI			SLAQ	
#	Descriptor	Definition	#	Symptom
1	Seizure	Recent onset. Exclude metabolic, infectious, or drug cause.	2p	Seizure
3	Organic brain syndrome	Altered mental function with impaired orientation, memory, or other intelligent function, with rapid onset fluctuating clinical features. Include clouding of consciousness with reduced capacity to focus and inability to sustain attention to environment, plus at least two of the following: perceptual disturbance, incoherent speech, insomnia or daytime drowsiness, or increased or decreased psychomotor activity. Exclude metabolic, infectious, or drug causes.	2r	Forgetfulness
6	Lupus headache	Severe persistent headache may be migrainous, but must be nonresponsive to narcotic analgesia.	2t	Unusual headaches
7	CVA	New onset of cerebrovascular accident(s). Exclude arteriosclerosis.	2q	Stroke
8	Vasculitis	Ulceration, gangrene, tender finger nodules, periungual, infarction, splinter hemorrhages, or biopsy or angiogram proof of vasculitis.	2m	Fingers/toes turning dead white or very pale in the cold
9	Arthritis	More than 2 joints with pain and signs of inflammation (i.e., tenderness, swelling, or effusion).	2w	Pain or stiffness in the joints
			2x	Swelling in the joints
10	Myositis	Proximal muscle aching/weakness, associated with elevated creatinine phosphokinase/aldolase or electromyogram changes or a biopsy showing myositis.	2u	Muscle pain
			2v	Muscle weakness
15	New Rash	New onset or recurrence of inflammatory type rash.	2f	Other rash
16	Alopecia	New onset or recurrence of abnormal, patchy, or diffuse loss of hair.	2i	Bald patches on scalp
17	Mucosal Ulcers	New onset or recurrence of oral/nasal ulcerations.	2d	Sores in mouth/nose
18	Pleurisy	Pleuritic chest pain with pleural rub or effusion or pleural thickening.	2k	Shortness of breath
			2l	Chest pain on deep breath
19	Fever	>38°C. Exclude infectious cause.	2c	Fevers (>101°F, 38.5°C) taken by thermometer

SLAQ versus SLEDAI: the description and definition of SLEDAI indices and the matched SLAQ symptoms.
